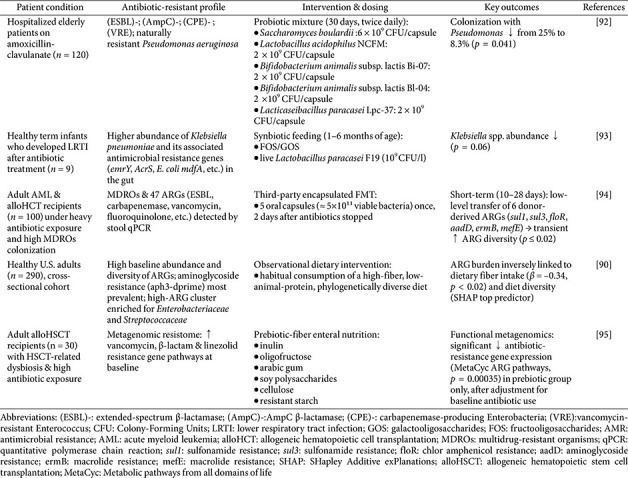# Microecological Interventions against Antibiotic-Induced Dysbiosis and Related Resistome Expansion

**DOI:** 10.4014/jmb.2601.01009

**Published:** 2026-03-26

**Authors:** Yuxuan Shi, Huajun Li

**Affiliations:** 1 The First Affiliated Hospital, Dalian Medical University, Dalian, Liaoning Province, P.R. China; 2 Department of Pathogen Biology and Microecology, College of Basic Medical Sciences, Dalian Medical University, Dalian, Liaoning Province, P.R. China

**Keywords:** Gut microbiota, Gut microbiota dysbiosis, Antibiotic resistance, Microbial metabolites, Microecological interventions

## Abstract

Antibiotic exposure and the emergence of antimicrobial resistance are critical global health threats, with antibiotic-induced gut dysbiosis contributing to increased mortality, prolonged illness, and significant economic burden. This review introduces the complex interplay between antibiotic exposure, gut microbiota dysbiosis, and the dissemination of antimicrobial resistance genes, which collectively undermine intestinal barrier function and promote systemic inflammation. It also explores how microbial metabolites influence resistance mechanisms through metabolic regulation, alteration of bacterial communities, antibiotic biotransformation, biofilm formation, and host–microbe interactions. Microecological interventions—including probiotics, prebiotics, synbiotics, postbiotics, fecal microbiota transplantation, dietary modifications, and emerging strategies—have the potential to restore microbial homeostasis, enhance colonization resistance to invading pathogens, and mitigate the spread of resistant pathogens. By integrating ecological and therapeutic perspectives, these approaches offer a sustainable framework for combating antibiotic resistance and improving clinical outcomes.

## Introduction

The human gastrointestinal tract constitutes a complex micro-ecosystem dominated by bacteria but also comprising fungi, viruses and archaea [1]. Bacterial communities exhibit marked compositional heterogeneity, with Firmicutes, Bacteroidetes, Actinobacteria and Proteobacteria representing the most prevalent phyla [2]. The spatial distribution and relative abundance of these taxa are profoundly altered by antibiotic exposure. For example, antibiotic treatment commonly decreases the relative abundances of Firmicutes and Bacteroidetes and shifts the Firmicutes/Bacteroidetes ratio downward, inducing intestinal dysbiosis [3]. Antibiotic resistance arises from adaptations that allow microbes to evade the effects of specific antibiotics through diverse mechanisms, rendering them incapable of being suppressed or killed by these drugs. This phenomenon has impacted the global health system, making the treatment of bacterial infections more challenging and complex [4]. According to a 2025 report by the World Health Organization (WHO) Global Antimicrobial Resistance and Use Surveillance System (GLASS), gram-negative bacteria such as *Escherichia coli* and *Klebsiella pneumoniae* are resistant to fluoroquinolones and third-generation cephalosporins, which were once first-choice treatments for these infections. In 2023, resistance to third-generation cephalosporins was reported in 44.8% (95% credible interval: 39.3, 50.4) of infections with *E. coli* and 55.2% (48.5, 61.7) of those with *K. pneumoniae*. Within the WHO African Region, the extent of resistance was especially concerning, exceeding 70% for both pathogens. The percentage resistance of *Staphylococcus aureus* bloodstream infections to methicillin was 27.1% (23.5, 31.0) globally, and was highest in the Eastern Mediterranean Region, at 50.3% (39.8, 60.8) in 2023. Since 2013, the global prevalence of severe multidrug-resistant (MDR) bacterial infections has exhibited a sustained upward trajectory [5], with Methicillin-Resistant *Staphylococcus aureus* (MRSA) causing over 100,000 deaths, with a death rate of 10.3 per 100,000 worldwide in 2019 [6]. Resistance is not limited to bacteria but also includes viruses, fungi, and parasites, collectively referred to as antimicrobial resistance (AMR) [7]. The World Bank warns that AMR poses a dual economic threat: cumulative global healthcare spending could rise by up to US$1 trillion by 2050, driving annual gross domestic product (GDP) losses ranging from US$1 trillion to US$3.4 trillion as soon as 2030. Strategies to prevent and treat gut microbial dysbiosis are of great importance. Antibiotic administration is a common trigger, disrupting the homeostasis of the gut microbiota. This disturbance breaks the balance between beneficial and pathogenic intestinal microorganisms, altering microbial metabolic activities and spatial distribution, and ultimately resulting in reduced microbial diversity, abnormal shifts in specific taxa, and dysfunction [8, 9].

In this review, we outline the current state of antibiotic resistance, examine how microbial metabolites influence antibiotic resistance, summarize current findings linking antibiotic resistance to disease, and focus on microecological interventions and therapeutic strategies to address antibiotic resistance.

## Antibiotic Resistance

Antibiotic resistance is spreading rapidly worldwide, contributing to an estimated 4.95 million deaths annually-1.27 million of which are directly attributed to resistant pathogens [6]. This crisis is no longer confined to hospital-acquired infections; it now permeates community settings, animal populations, and the broader environment [10]. Antibiotic resistance is the acquired ability of bacteria to survive and continue growing despite exposure to an antimicrobial agent at concentrations that would normally inhibit or kill them. This renders standard treatments ineffective and leads to prolonged illness, higher healthcare costs, and increased mortality. Fungi, parasites, and viruses can also develop drug resistance. The ESKAPE group of pathogens, namely *Enterococcus faecium*, *S. aureus*, *K. pneumoniae*, *Acinetobacter baumannii*, *Pseudomonas aeruginosa*, and *Enterobacter* spp., have emerged as formidable MDR strains that now dominate clinical treatment challenges [11].

One key way that bacteria develop drug resistance is through genetic mutation. These mutations can occur either naturally or adaptively in response to antibiotic selection pressure. Gut bacteria acquire resistance by accumulating spontaneous genetic mutations that alter β-lactamase gene activity or modify ribosomal structure [12]. Under the selective pressure of antibiotics, gut bacteria may undergo adaptive mutation that enhances their likelihood of surviving treatment [13]. Horizontal gene transfer (HGT) refers to the direct transfer of genetic material between different microorganisms [14]. This mechanism is particularly prevalent among microbes residing in the gut, which represents a complex microbial ecosystem. Interactions between bacteria and their host, such as nutrient availability and immune pressures, create an ideal environment for HGT, facilitating the rapid dissemination of antibiotic-resistance genes (ARGs) throughout the microbial population. For instance, in mice treated with ampicillin, *E. coli* O80:H26 was shown to transfer its β-lactamase gene *blaCMY-2* to *Salmonella enterica* serovar Heidelberg through conjugation [15]. The widespread use of antibiotics exerts strong selection pressure on microbial communities. A healthy gut is characterized by high microbial diversity, which helps resist invasion by pathogens and the disruptive effects of antibiotics [16]. The normal gut microbiota also serves as a significant reservoir of ARGs. Antibiotic exposure can alter the composition of the indigenous microbial community in the host, promoting the growth of antibiotic-resistant bacteria (ARB) that may subsequently cause opportunistic infections [17].

## Gut microbiota Dysbiosis

### Distinction between Microbiota Dysbiosis and ARG Dissemination

Gut microbiota dysbiosis is defined as an imbalance of the gut microbial community. This imbalance involves an increase in the abundance of pathogens alongside decreases in overall microbial diversity and the abundance of beneficial and keystone microbes of the core microbiota, which play vital roles in its ecological structure and function [18]. The gut resistome represents the combined abundance of all ARGs circulating in the gut, which determines how an individual’s gut microbiome will respond to and recover from antibiotic therapy [19]. Antibiotic administration induces gut microbiota dysbiosis, creating ecological opportunities for ARG enrichment and horizontal transfer that expand the gut resistome [20].

### Impaired Intestinal Barrier Function

Dysbiosis of the gut microbiota compromises intestinal barrier integrity, leading to increased intestinal permeability [21]. This allows the translocation of noxious agents (*e.g.*, bacterial toxins and metabolites) into the circulation, triggering systemic inflammation and immune responses. A mouse study demonstrated that antibiotic intervention with ceftriaxone induced prominent ileal mucosal edema and loss of epithelial integrity, simultaneously downregulating the tight-junction proteins ZO-1, Occludin, and Claudin-4, and markedly increasing the paracellular permeability of the intestinal epithelium [22].

### Activation of Inflammatory Responses

Gut microbiota dysbiosis can activate the host's inflammatory response [23]. One study showed that 4 weeks of orally administered vancomycin or polymyxin B in C57BL/6 mice caused significant gut dysbiosis characterized by reduced alpha-diversity and activated inflammatory pathways. Under lipopolysaccharide (LPS) stimulation, mouse splenocytes exhibited statistically significant upregulated expression of both tumor necrosis factor-alpha (TNF-α) and interferon-gamma (IFN-γ) [24]. Obesity or gut microbiota dysbiosis can lead to an elevated Firmicutes/Bacteroidetes ratio, which is accompanied by increased abundance of LPS-producing bacteria [25, 26]. LPS potentiates the expression and release of key pro-inflammatory cytokines, including TNF-α and interleukin (IL)-6, leading to a state of chronic low-grade systemic inflammation [27].

## Inflammatory Bowel Disease (IBD)

### Impact of Antibiotic Use on the Gut Microbiota in IBD

Studies in patients with IBD indicate that antibiotic exposure alters the gut microbial composition, notably diminishing beneficial bacterial populations, and that the microbiota gradually re-establishes a new homeostatic state after treatment discontinuation [28]. According to a Danish nationwide cohort study, antibiotic administration significantly increases the risk of developing IBD across all age groups, with the highest risk observed in individuals over 40 years (incidence rate ration: ≤ 1.48). There is a clear dose–response relationship, with those receiving five or more antibiotic courses facing nearly double the risk. The risk of IBD onset is highest within 1–2 years after antibiotic exposure and declines thereafter. Antibiotics used to treat gastrointestinal pathogens such as nitroimidazoles and fluoroquinolones carry the greatest risk. The Danish study highlights that antibiotics disrupt the gut microbiota by depleting beneficial bacteria and increasing potentially harmful species, leading to long-lasting dysbiosis that may trigger or exacerbate IBD [29].

### Dissemination of ARGs

The spread of ARGs is pronounced in patients with IBD [30]. For instance, the intestinal microbiota of Crohn’s disease patients exhibits enrichment of *E. coli* strains that carry multiple ARGs involved in efflux pumps and express virulence factors characteristic of invasive pathovars [31]. Furthermore, the horizontal transfer of ARGs among species, especially between taxonomically related microbes, rises sharply following antibiotic exposure. This dissemination further complicates the management of IBD patients.

### Influence of Resistant Pathogens

The emergence of specific resistant pathogens pose additional therapeutic challenges in IBD patients. For example, *Clostridioides difficile* infections are relatively common in IBD, and their high level of antibiotic resistance is especially prominent in pediatric populations [32]. Additionally, MDR *E. coli* is more prevalent among patients with ulcerative colitis compared to healthy controls, likely driven by selective pressures within the inflamed intestinal milieu [33]. These resistant pathogens not only heighten therapeutic complexity but may also exacerbate disease severity.

## Fungal Infections

Fungal infections, particularly those caused by *Candida* species, have become a major global health burden. Invasive candidiasis accounts for approximately 70% of all invasive fungal infections (IFIs) worldwide and carries a mortality rate of up to 50% [34]. In recent years, infections with *Candida* species other than *C. albicans* (*e.g.*, *C. auris* and *C. glabrata*) have increased; these strains often exhibit higher levels of resistance, further complicating treatment [35, 36]. Prolonged antibiotic use disrupts the equilibrium of the gut microbiota, mainly by diminishing beneficial bacterial populations, thereby weakening the intestine’s natural antifungal defenses. Studies have shown that antibiotics suppress lymphocyte-dependent antifungal immunity mediated by IL-17A and granulocyte–macrophage colony-stimulating factor, increasing susceptibility to invasive *Candida* infections. This immunosuppression can enable noninflammatory translocation of intestinal bacteria and systemic bacterial coinfection, exacerbating the risk of fungal disease [37]. In clinical studies, antibiotic exposure is significantly associated with the incidence of IFI. For example, a multicenter prospective case–control study found that, among preterm or very low birthweight infants, both the antibiotic utilization rate and the number of days of therapy in the 4 weeks preceding IFI were significantly linked to the development of infection. Use of third-generation cephalosporins and carbapenems, in particular, has been identified as a major risk factor for IFI [38].

## Effect of Microbial Metabolites on Antibiotic Resistance

### Metabolic Regulation

The gut microbiota produces diverse metabolites that regulate metabolic pathways in both hosts and bacteria [39]. For example, certain short-chain fatty acids (SCFAs) can enhance the intestinal chemical barrier by stimulating the secretion of antimicrobial peptides and mucins, indirectly reducing the colonization and transmission of drug-resistant bacteria [40, 41]. *A. muciniphila* is a mucin-degrading, strictly anaerobic, gram-negative bacterium belonging to the Verrucomicrobia phylum. First isolated and described in 2004, it is a common resident of the human intestinal tract, typically constituting 1%–4% of the gut microbiota in healthy adults. *A. muciniphila* colonizes the outer mucus layer of the intestinal epithelium, where it utilizes mucin glycoproteins as its primary carbon and nitrogen source [42]. Currently, it is primarily defined as a next-generation beneficial microbe, acting as a key player in maintaining intestinal mucus layer homeostasis and gut barrier integrity [43].

**Fig. 1:**
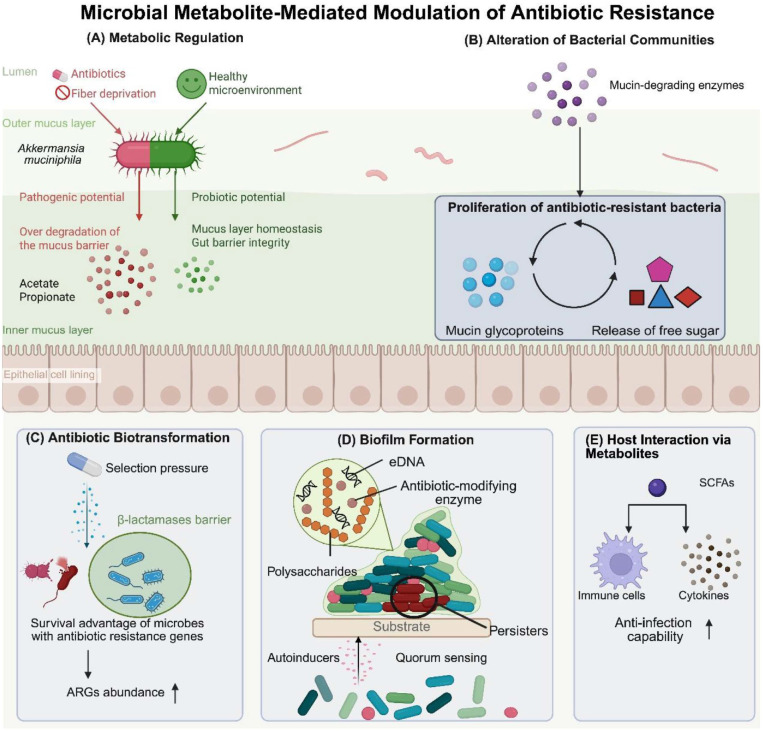
Pathways of microbial metabolite-mediated regulation of antibiotic resistance. (**A**) Metabolic Regulation: Antibiotics and fiber deprivation in the lumen make *Akkermansia muciniphila* a pathogenic bacterium. The excessive degradation of mucin paves the way for the invasion of resistant pathogens, which then thrive on the metabolites derived from *A. muciniphila*. In contrast, *A. muciniphila* residing in a healthy microenvironment enhances gut barrier integrity and protects against antibiotic-resistant microbes. (**B**) Alteration of Bacterial Communities: Metabolites reshape microbial composition through competitive inhibition and signaling, often favoring resistant bacteria. *A. muciniphila* degrades mucin glycoproteins via mucin-degrading enzymes, releasing free sugars that promote proliferation of antibiotic-resistant strains. (**C**) Antibiotic Biotransformation: Antibiotics kill microbes lacking resistance genes. Hydrolytic enzymes secreted by antibiotic resistant microbes such as β-lactamases form barriers against antibiotics. (**D**) Biofilm Formation: Secondary metabolites autoinducers-mediated quorum sensing regulates biofilm formation. The composition of biofilm includes polysaccharides, protein, and eDNA, which plays a significantly important role in antibiotic resistance. (**E**) Host interaction via metabolites: SCFAs enhances anti-inflammatory signaling and cytokine production, strengthening immune clearance of pathogens and indirectly reducing selective pressure for resistance. (Created with BioRender.com.)

Many gut microbes that cause disease under certain genetic or environmental conditions can also be protective within a specific surrounding community or in the context of infection with an overt pathogen [44]. For example, the same *Ruminococcus gnavus* strains that act as inflammatory triggers in genetically susceptible hosts also serve as defenders against pathogen invasion or epithelial breach when the microbial community or infectious context changes [45]. In mice, fiber deprivation creates mucus utilization stress. Under this stress, *A. muciniphila* over-degrades the mucus barrier, inadvertently acting as an accomplice to pathogen invasion and markedly increasing susceptibility to infection and mortality [46]. The regulatory influence of *A. muciniphila* on antibiotic resistance is also a double-edged sword, showing strong dependence on features of the human gut microenvironment, including antibiotic exposure, nutrition, host genetics, and interactions with surrounding microbes (Fig. 1) [47]. In a healthy intestinal microenvironment, *A. muciniphila*-mediated mucin degradation primarily produces two SCFAs: acetate and propionate. These metabolites serve as crucial energy sources for colonocytes and act as regulators of systemic host metabolism. In contrast, the reduced diversity of gut microbes in an antibiotic-disturbed, imbalanced intestine confers a prominent competitive advantage to drug-resistant pathogens. Under such circumstances, the nutrients derived from mucus degradation by *A. muciniphila* are more readily sequestered and utilized by these resistant pathogens. Additionally, antibiotic exposure can induce promoter mutations in the TEM-type β-lactamase gene in this bacterium, which not only enhances its own drug resistance but also leads to the loss of its host-protective effects against diet-induced obesity [48].

### Alterations in Bacterial Communities

The composition of the gut microbiota modulates antibiotic therapeutic efficacy through interspecies interactions and competitive dynamics. Specific microbial metabolites may induce selective pressure that favors the proliferation of resistant bacteria while suppressing susceptible strains, ultimately increasing the relative abundance of ARB. This is exerted through competitive inhibition or the release of signaling molecules [49]. For instance, *A. muciniphila* has been shown to structurally remodel gut microbial communities through its metabolic activity. The mucin-degrading enzymes produced by this species catalyze the hydrolysis of intestinal mucin glycoproteins, liberating bioavailable nutrients that preferentially sustain the proliferation of ARB [50].

### Biotransformation of Antibiotics

Intestinal microorganisms can alter the chemical properties of antibiotics through their metabolic activities, affecting their efficacy. For instance, certain bacteria directly inactivate antibiotics through enzymatic decomposition or chemical modification [51]. The overall abundance and diversity of ARGs were reportedly significantly lower in adults and infants without recent antibiotic use compared with those with a recent history of antibiotic exposure. Following antibiotic withdrawal, overall ARG abundance showed a marked temporal decline [52]. The selection pressure of ng/kg-level residual antibiotics has been shown to alter the type of ARGs and increase their abundance in the human gut [53]. Bacteria capable of producing antibiotic-inactivating enzymes (*e.g.*, β-lactamases) can neutralize these drugs, gaining a survival advantage. Consequently, antibiotic selection pressure favors the proliferation of strains possessing the respective ARGs. Therefore, dysbiosis signifies not merely a change in microbial composition, but a shift in the functional capabilities of the entire microbial community. This enriched transforming capacity establishes an enzyme barrier, effectively lowering antibiotic concentrations within the gut. Importantly, this capacity is more than just a static metabolic trait; it is a dynamic ecological function that protects resistant bacteria while potentially creating a niche for pathogens or opportunistic bacteria, thereby exacerbating infections and contributing to therapeutic failure.

### Biofilm Formation

Biofilms are communities of microbial cells that attach to surfaces and become embedded in a self-produced extracellular matrix, making them far more resistant to antimicrobial agents than their free-floating, planktonic counterparts [54]. Biofilm formation is synergistically enhanced through the convergence and mutual regulation of extracellular quorum sensing and intracellular cascades of cyclic dinucleotide signaling. Quorum sensing is a vital intercellular communication mechanism that enables bacteria to detect population density via specific signaling molecules called autoinducers. These secondary metabolites include N-acyl homoserine lactones (AHLs) in gram-negative bacteria, autoinducing peptides (AIPs) in gram-positive bacteria, and autoinducer-2 (AI-2), which functions across both groups (Table 1). The antibiotic resistance of biofilms stems from characteristic features including restricted antimicrobial penetration, efflux pumps, antibiotic-modifying enzymes within the matrix, extracellular DNA (eDNA), persister cells, and HGT [55].

### Interactions between Microbial Metabolites and the Host

Gut microbiota metabolites can affect the bacteria themselves but also indirectly influence antibiotic efficacy by modulating the host immune response. Some metabolites enhance the host immune response, aiding in the elimination of infecting bacteria, while others suppress the immune system, providing a growth advantage for ARB [61]. Metabolites produced by *Lactobacillus* and *Bifidobacterium*, such as SCFAs, can enhance the host immune response through their anti-inflammatory effects, aiding in the clearance of infecting bacteria. For instance, the SCFA butyrate modulates the activity of immune cells and the production of cytokines, improving the host's anti-infection capability [62].

## Microecological Interventions for Antibiotic Resistance

**Fig. 2:**
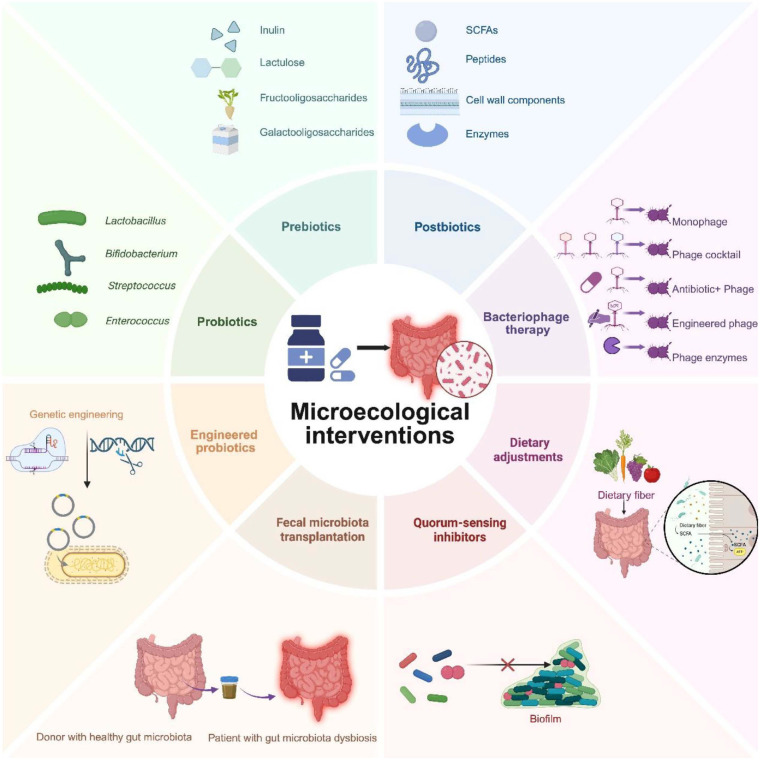
Microecological interventions for antibiotic resistance and gut microbiota dysbiosis. Microecological interventions are designed to target the intestinal microecology, comprising probiotics, prebiotics, synbiotics (the combination of probiotics and prebiotics), postbiotics, FMT, dietary adjustments, engineered probiotics, bacteriophage therapy, and quorum-sensing inhibitors. (Created with BioRender.com.)

Contemporary therapeutics aimed at countering antibiotic resistance work by restoring balance to the composition and function of the gut microbiota (Fig. 2). With the rapid advances in gut microecology, a variety of microecological interventions are becoming widely used to mitigate antibiotic resistance, as supported by human studies (Table 2).

### Probiotics and Prebiotics

Probiotics are live, non-pathogenic microorganisms that restore intestinal microbial homeostasis, conferring measurable health benefits on the host when administered in adequate quantities [63]. The most clinically well-documented probiotic taxa belong to *Lactobacillus* and *Bifidobacterium* genera [64]. Probiotics significantly lower the abundance of ARGs by reshaping the gut microbiota. Specific strains have been shown to reduce the absolute levels of tetracycline resistance genes, including *tetM* and *tetO*, and these reductions are negatively correlated with the abundance of *Bifidobacteriaceae* [65]. Furthermore, probiotic-driven feed fermentation in pigs—for example, with *Weissella*—improved growth performance and decreased the prevalence of multiple ARGs (*e.g.*, *tetX*, *tetW*, and *ermG*) in feces [66].

Prebiotics are nondigestible dietary constituents, exemplified by fructooligosaccharides and galactooligosaccharides, that selectively stimulate the growth and metabolic activity of beneficial gut bacteria such as *Bifidobacterium* [67]. These effects not only reshape the intestinal microbial community but also fortify the epithelial barrier, attenuating pathobiont colonization [68]. Specific prebiotics, such as xylo-oligosaccharides have been shown to significantly increase the abundance of *Bifidobacterium* in the gut, thereby inhibiting the growth of drug-resistant bacteria [69]. By enhancing the host's natural immune defenses and improving gut health, prebiotics can reduce the incidence of infections, decreasing reliance on antibiotics. Reducing antibiotic use not only helps slow the development of antibiotic resistance but also minimizes the side effects associated with overuse [70].

### Synbiotics

Synbiotics, which combine probiotics and prebiotics, leverage their synergistic interactions to effectively restructure the gut microbiota, selectively enriching beneficial taxa while suppressing the proliferation of pathobionts [71]. For instance, under simulated gastrointestinal conditions, a synbiotic formulation comprising *Limosilactobacillus reuteri*, *Bifidobacterium longum*, and galactooligosaccharides demonstrated high viability and significantly elevated the relative abundances of *Lactobacillus* and *Bifidobacterium*, reducing populations of *Lachnoclostridium* and other potentially harmful microbes [72]. Synbiotics restore gut microbial balance by simultaneously supplying beneficial bacteria—such as lactobacilli and bifidobacteria—and the prebiotics that fuel their growth. This rebalanced ecosystem exhibits reduced pathogen colonization and proliferation, diminishing the need for antibiotic therapy [73, 74]. A study has shown that dysbiosis of the gut microbiota is closely linked to the dissemination of ARGs, and that synbiotics can attenuate ARG abundance by rebalancing the microbial community [65]. Synbiotics can also reduce antibiotic-associated adverse effects, such as antibiotic-associated diarrhea (AAD), helping to improve patient adherence to antibiotic therapy and curb the development of resistance [74]. In clinical studies, synbiotics maintained intestinal microbial balance and significantly lowered both the incidence and severity of AAD [75].

### Postbiotics

Unlike probiotics, postbiotics contain no live microorganisms and therefore cannot disseminate ARGs [76, 77]. Postbiotics are defined as non-viable microbial constituents or metabolic products generated through probiotic fermentation, encompassing SCFAs, enzymes, vitamins, cell-wall fragments, and other bioactive molecules [78]. These bioactive constituents show health benefits independent of viable microorganisms, offering pronounced advantages in safety, stability, and application breadth [79]. Certain postbiotic components, such as bacteriocins and SCFAs, exhibit direct antimicrobial activity, inhibiting or even killing drug-resistant pathogens. For example, bacteriocins produced by lactic acid bacteria can disrupt the cell membranes of pathogenic organisms, suppressing their growth [80]. Postbiotics modulate the balance of the gut microbiota, curbing the colonization and proliferation of resistant pathogens. By promoting the growth of beneficial bacteria while suppressing pathogenic species, they diminish the risk of antibiotic resistance transmission [81]. As an alternative or adjunctive therapy, postbiotics can also lessen reliance on antibiotics and inhibit the emergence and spread of AMR [82].

### Fecal Microbiota Transplantation (FMT)

FMT is an innovative therapeutic approach that transfers the complete gut microbiota from a healthy donor into the gastrointestinal tract of a recipient with dysbiosis [83, 84]. FMT greatly enhances gut microbial diversity, particularly in patients whose microbiota has been depleted by antibiotic exposure, chronic disease, or infection with *C. difficile*, while restoring microbiome functionality and strengthening colonization resistance against pathogens [85, 86]. This approach has achieved notable success in treating infections caused by MDR organisms. For example, studies on carbapenemase-producing Enterobacteriaceae have shown that FMT markedly reduces their colonization rates and improves clinical outcomes [87]. One study indicated that FMT rapidly lowers the abundance of AMR genes by introducing a healthy donor microbiota, and over the longer term it suppresses both their transmission and expression by reestablishing a stable gut microbial community [88].

### Dietary Adjustments

The gut microbiome serves as a major reservoir of ARGs, and dietary factors can directly shape ARG abundance and diversity by altering microbial composition and function [89]. Epidemiological studies show that high-fiber, diversified diets are associated with a lower ARG carriage rate. For example, increased intake of whole grains and dietary fiber fosters the expansion of beneficial taxa such as *Clostridiaceae*, diminishing the presence of ARGs [90]. Dietary adjustments, particularly increased fiber intake, have also been shown to significantly ameliorate antibiotic-induced gut microbiota dysbiosis, in part by modulating intestinal redox potential [91]. Dietary fiber is the primary energy source for beneficial gut microbes, promoting the production of SCFAs that suppress pathogen growth and inhibit the spread of ARGs. Incorporating whole grains, vegetables, fruits, and legumes into the daily diet is therefore recommended to boost fiber intake (Table 2).

## Major Limitations of Clinical Evidence for Microecological Interventions

Table 1 summarizes key clinical evidence supporting various gut microbiota-targeted interventions. When interpreting these positive findings, however, it is crucial to consider methodological limitations and clinical heterogeneity. Below, we offer an in-depth critique of these aspects.

Despite promising trends shown in multiple studies, significant heterogeneity in probiotic strain, dosage, duration, administration route, and patient baseline characteristics hinders the formulation of unified clinical recommendations. Most studies highlighted the importance of specific probiotic strains [96]. Strains of *Lactobacillus* and *Bifidobacterium*, which were identified as notably effective in adult populations, appear to restore gut microbiota balance and thereby help to mitigate antibiotic resistance [97]. Patient baseline characteristics, such as geographical regions and diets, are also important sources of heterogeneity. Mediterranean, Japanese, Korean, plant-based, calorie-restricted, high-fiber, and low-protein diets have all been associated with increased abundances of SCFA- and lactic acid-producing bacteria, as well as decreased abundances of opportunistic pathogenic bacteria. By contrast, Western, animal-based, low short-chain carbohydrate, and gluten-free diets have been linked with reduced levels of SCFA-producing bacteria [98].

Most of the cited studies are limited by a small sample size (n < 100), resulting in insufficient statistical power. This limitation may hinder the detection of small yet clinically meaningful differences and potentially contribute to spuriously positive results. Furthermore, data on long-term efficacy are severely lacking. For example, the long-term effect of FMT remains inadequately deﬁned [99].

Although FMT is generally considered safe, reported adverse events include transient diarrhea, abdominal cramps or pain, low-grade fever, bloating, flatulence, and constipation. Of particular concern is the potential transmission of infections from donor stool, highlighting the need for rigorous donor screening and testing for MDR microbes prior to FMT [100]. Furthermore, mobile genes—particularly ARGs from probiotic bacteria—can be transmitted to other bacteria within the intestinal tract via HGT, including transformation, conjugation, or transduction. If disease-causing pathogens acquire these ARGs, the effectiveness of antibiotic therapy for consumers may be reduced [101].

## Emerging Strategies of Microecological Interventions

### Engineered Probiotics

Considering the uncertainties surrounding the long-term efficacy of probiotic intervention and the risk of HGT, advances in gene sequencing and genome editing technologies have made it feasible to bioengineer novel probiotics capable of eliminating ARB-mediated infections. There are two primary strategies to synthetic biology approaches using living organisms: top-down, which involves genome reduction to delete nonessential genes, and bottom-up, which involves genome synthesis to add essential genes [102]. Probiotics can be genetically tailored to improve their function and targeted delivery by enhancing mechanisms such as toxin neutralization, expression of specific antimicrobial peptides, antibody production, replacement intervention, detoxification, adjustment of the immune system, and enzyme supplementation [103].

### Bacteriophage Therapy

The shortage of new antibiotics and the rise of MDR bacterial strains have highlighted bacteriophage (BP) or phage therapy as an alternative. BPs are viruses that naturally infect and kill their host bacteria, exhibiting greater target specificity than antibiotics without directly infecting or damaging human cells. BPs can eradicate the target pathogenic bacterium without altering the commensal microflora, pointing to an obvious advantage of this therapy [104]. There are four main types of resurgent BP therapy. Conventional phage therapy uses lytic BPs, either as monophages or phage cocktails, to eliminate specific bacterial infections. Combinatorial phage therapy employs a combination of BPs and antibiotics to create “phage–antibiotic synergy” (PAS). Phage-derived lytic enzymes, including lysins and holins, are also widely used to treat infections with ARB. Lastly, bioengineered phages, including fusions of gram-positive lysin phages, are designed to release novel lysins for improved antimicrobial efficacy [105].

### Quorum-Sensing Inhibitors

Quorum sensing, a form of intercellular communication mediated by autoinducers, is an essential step in biofilm formation. Quorum-sensing inhibitors serve as a promising alternative to traditional antibiotic therapy and may reduce the development of MDR. For example, meta-bromo-thiolactone (mBTL) is a potent quorum and virulence inhibitor of *S. aureus* and MRSA. A study describing the development of mBTL-loaded chitosan nanoparticles reported reductions of 51.7% in wild-type *S. aureus* and 50% in MRSA for biofilm formation [106].

## Conclusion

Antibiotic resistance and the consequent disruption of gut microbial homeostasis represent interconnected facets of a global health crisis. This review has delineated the complex pathways through which antibiotic exposure drives dysbiosis, undermines intestinal barrier integrity, and fuels the selection and horizontal transfer of ARGs. Furthermore, we have explored how this dysregulated state contributes to the pathogenesis and complications of conditions ranging from IBD to IFIs.

Crucially, the evidence compels a paradigm shift away from viewing antimicrobial therapy in isolation and toward an ecological perspective that includes the host microbiome. Microecological interventions, including probiotics, prebiotics, synbiotics, postbiotics, FMT, dietary modifications, and emerging strategies, are not merely supportive adjuncts but rather offer fundamental approaches to prevent, mitigate, and reverse AMR. By restoring microbial diversity, strengthening colonization resistance, directly antagonizing pathogens, and modulating host immunity, they can reduce the selective pressure that fosters resistant strains. The most effective intervention is to prevent the development of antibiotic resistance and dysbiosis. This requires a proactive approach that views antibiotics as a precious public resource, whose effectiveness must be maintained through collective action on a global scale. Antibiotic stewardship refers to coordinated interventions within healthcare facilities and communities to promote the appropriate use of antibiotics, achieving optimal patient outcomes while minimizing adverse effects and AMR. Its core principle is “the right drug, at the right dose, for the right duration, at the right time.” Regulatory oversight is a powerful driver and safeguard for antibiotic stewardship programs.

Although these interventions show promise in combating AMR, their extensive application in clinical practice should be preceded by further research to tackle key challenges Additionally, a shift towards personalized and precise treatment, centered on a dynamic and comprehensive assessment of each patient's individual status, is an inevitable direction and key strategy for addressing AMR. The fundamental breakthrough of this approach lies in its treatment goal, which expands beyond merely killing pathogenic bacteria to restoring and maintaining the healthy homeostasis of the host–microbe–environment triad. Achieving this necessitates viewing the patient as a dynamic, complex system encompassing multiple variables. Another key challenge is the therapeutic evaluation of microecological interventions in large-scale, long-term clinical trials designed to assess both efficacy and safety.

## Figures and Tables

**Table 1:** Key secondary metabolites as quorum-sensing autoinducers and their roles in biofilm regulation.

secondary metabolites	Bacterial species	Key pathways	Role in biofilm regulation	References
AI-2	*Streptococcus suis*	LuxS/AI-2 quorum sensing system	Significantly promotes biofilm formation and enhance bacterial antibiotic resistance	[56]
Surfactin	*Bacillus subtilis*	• *tapA-sipW-tasA* operon • *epsA-O* operon	Induces the biosynthesis of the extracellular matrix	[57]
AHLs	• *Klebsiella pneumoniae* • *Enterobacter* • *Citrobacter amalonaticus*	AHL-mediated quorum sensing system	Regulates the composition, metabolic function, and pathogenic potential of oral biofilms through oxygen-dependent condition.	[58]
AIP	*Staphylococcus aureus*	Agr quorum sensing system	Orchestrates biofilm dispersion	[59]
AHLs	*Pseudomonas aeruginosa* (PAO1 strain)	• LasR-LasI quorum sensing system • RhlR-RhlI quorum sensing system	Regulates release of eDNA and biofilms structure	[60]

Abbreviations: AI-2: Autoinducer-2; AHLs: N-acyl homoserine lactones; AIP: autoinducer peptide

**Table 2:** Microecological interventions for antibiotic resistance on human.